# Computerized System for Staging Peritoneal Surface Malignancies

**DOI:** 10.1245/s10434-015-4966-5

**Published:** 2015-11-12

**Authors:** Paolo Sammartino, Daniele Biacchi, Tommaso Cornali, Fabio Accarpio, Simone Sibio, Bernard Luraschi, Alessio Impagnatiello, Angelo Di Giorgio

**Affiliations:** Dipartimento di Chirurgia P. Valdoni, Università di Roma Sapienza, Rome, Italy

## Abstract

**Background:**

Peritoneal surface malignancies (PSMs) are usually staged using Sugarbaker’s Peritoneal Cancer Index (PCI) and completeness of cytoreduction score (CC-s). Although these staging tools are essential for selecting patients and evaluating outcome after cytoreductive surgery (CRS) plus hyperthermic intraperitoneal chemotherapy (HIPEC), both scoring models lack some anatomic information, thus making staging laborious and unreliable. Maintaining Sugarbaker’s original concepts, we therefore developed a computerized digital tool, including a new anatomic scheme for calculating PCI and CC-s corresponding closely to patients’ real anatomy. Our new anatomic model belongs in a web-based application known as the PSM Staging System, which contains essential clinical and pathological data for the various PSMs currently treated.

**Methods:**

The new digital tool for staging PSM runs on a personal computer or tablet and comprises male and female colored anatomic models for the 13 endoabdominal regions, with borders defined according to real anatomic landmarks. A drag-and-drop tool allows users to compute the PCI and CC-s, making it easier to localize and quantify disease at diagnosis and throughout treatment, and residual disease after CRS.

**Conclusions:**

Once tested online by registered users, our computerized application should provide a modern, shareable, comprehensive, user-friendly PSM staging system. Its anatomic features, along with the drag-and-drop tool, promise to make it easier to compare preoperative and postoperative PCIs, thus improving the criteria for selecting patients to undergo CRS plus HIPEC. By specifying the size, site, and number of residual lesions after CRS plus HIPEC, our digital tool should help stratify patients into outcome classes.

**Electronic supplementary material:**

The online version of this article (doi:10.1245/s10434-015-4966-5) contains supplementary material, which is available to authorized users.

 Peritoneal spread from an intraperitoneal neoplasia, or primary peritoneal tumors, currently identified as peritoneal surface malignancies (PSMs), are dismal events. Thanks to the pioneering efforts of Sugarbaker, their treatment has markedly improved results in the past 20 years.^1^,^2^ Standardizing procedures for surgical cytoreduction (peritonectomy procedures) associated with perioperative chemotherapy, combined with hyperthermia, hyperthermic intraperitoneal chemotherapy (HIPEC) can now guarantee hitherto unforeseeable results and quality of life in selected cases.^3^–^7^ In all malignancies, the extent of disease at diagnosis, and residual disease after treatment, are assessed with specific staging classifications. Staging systems provide the basis for defining which groups to include in clinical trials, and are the benchmark for evaluating patients’ outcome after treatment. For many years now, staging for patients with PSMs has mainly used two classification models, both developed and perfected by Sugarbaker.^8^ The first, the Peritoneal Cancer Index (PCI), assesses the extent of peritoneal disease at diagnosis and treatment. It quantitatively combines cancer implant size with tumor distribution throughout 13 abdominopelvic regions, producing a maximum score of 39. Two transverse and two sagittal straight lines, together with small bowel subdivision, artificially divide the abdomen into 13 regions (Fig. 1a). The second, the completeness of cytoreduction score (CC-s), analyzes the completeness of cytoreduction obtained by surgical procedures, and quantifies, from CC0 to CC3, eventual residual disease according to its size (Fig. 1b). Patients’ PSM outcome achieved with a combined treatment approach [cytoreductive surgery (CRS) plus HIPEC] correlates inversely with the extent of disease (PCI at diagnosis), and directly with the completeness of cytoreduction obtained at surgery. In specific clinical conditions, such as peritoneal metastases from colorectal and gastric cancer, the amount of peritoneal spread negatively influences the patient’s outcome to such an extent that the PCI score seems to acquire a specific role as a cut-off value for selecting candidates for CRS plus HIPEC.^6^,^9^–^12^ In these patients, only an extremely low PCI score and complete cytoreduction (CC0) allow long-term survival.^13^,^14^ In less aggressive PSM (low-grade appendiceal pseudomyxoma and peritoneal metastases from ovarian cancer), the major prognostic indicator seems to be the CC-s. Even in patients who have a high PCI, CRS plus HIPEC could achieve a good outcome provided that it leaves minimal residual disease.^3^,^15^

**Fig. 1:**
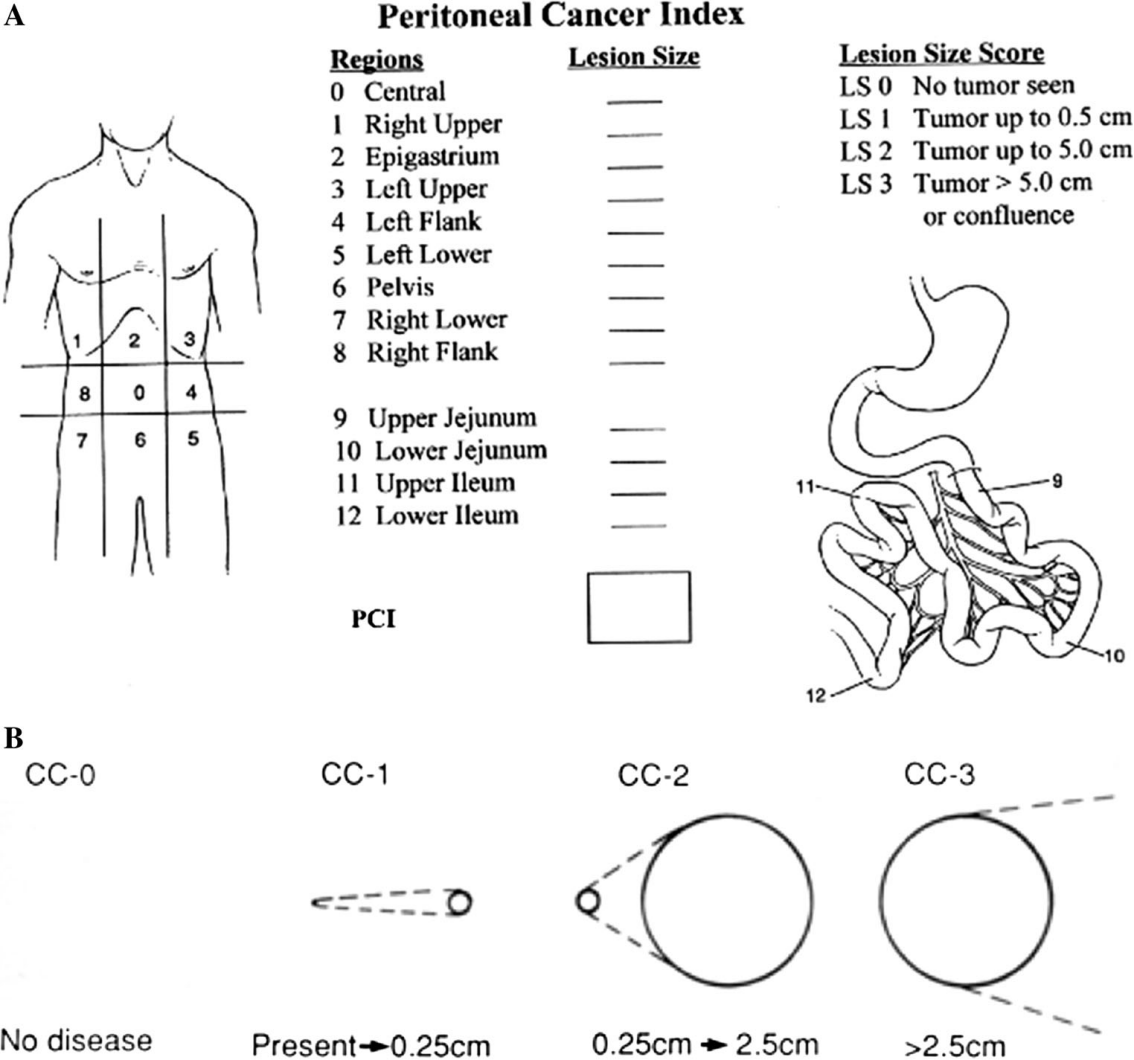
**a** PCI and **b** CC-s, according to Sugarbaker. *PCI* Peritoneal cancer index, *CC-s* completeness of cytoreduction score

The currently available PCI and CC-s classifications, according to Sugarbaker, leave room for improvement. The poorly informative two-dimensional anatomic model for scoring the PCI fails to reflect the patient’s real anatomy, as seen by the radiologist or surgeon. Although Sugarbaker himself later listed the anatomic structures involved in the 13 abdominopelvic regions,^16^ the model lacked depth, the anatomic structures contained in a specific abdominal region overlapped, and imaginary lines subdivided the regions. Hence, the problem remained unsolved. The PCI applied in this way therefore tends to make assessment more laborious and probably yields less reliable diagnostic and surgical findings on the extent of disease. Similar limitations apply to the CC-s because it completely lacks an anatomic model and therefore gives no information on the sites of residual disease or their number.

Despite keeping Sugarbaker’s original concepts, the foregoing shortcomings prompted us to facilitate PSM staging by optimizing the same anatomical model for applying the PCI and CC-s. With this concept in mind, we aimed to develop a computerized scheme containing images with a three-dimensional effect suitable for use during diagnosis or therapy and corresponding as closely as possible to the patient’s real anatomy. We included this model in a new web application known as the PSM Staging System, which contains the main clinical and pathological data for the various PSMs currently treated (www.psmss.net). The digital technology used in the new anatomic model should help localize and quantify, with greater precision, the extent of peritoneal disease (PCI) in diagnostic and surgical settings, specifying the size and number of lesions. Using the same anatomic model, the same digital tool also specifies the size and number of residual lesions after CRS, as well as the site of residual disease. Both user-friendly features aim to make the PCI and CC-s easier to mark and more reliable, therefore facilitating information exchange among physicians involved in treating PSMs. By providing a single web application for radiologists and surgeons, we also wanted to make it easier to compare preoperative and surgical disease staging, thereby improving patient selection criteria. With a more realistic anatomic model, we finally sought to extend current prognostic information correlating the PCI and CC-s according to the specific anatomic sites.

## Computerized Peritoneal Surface Malignancy Staging System

**Fig. 2:**
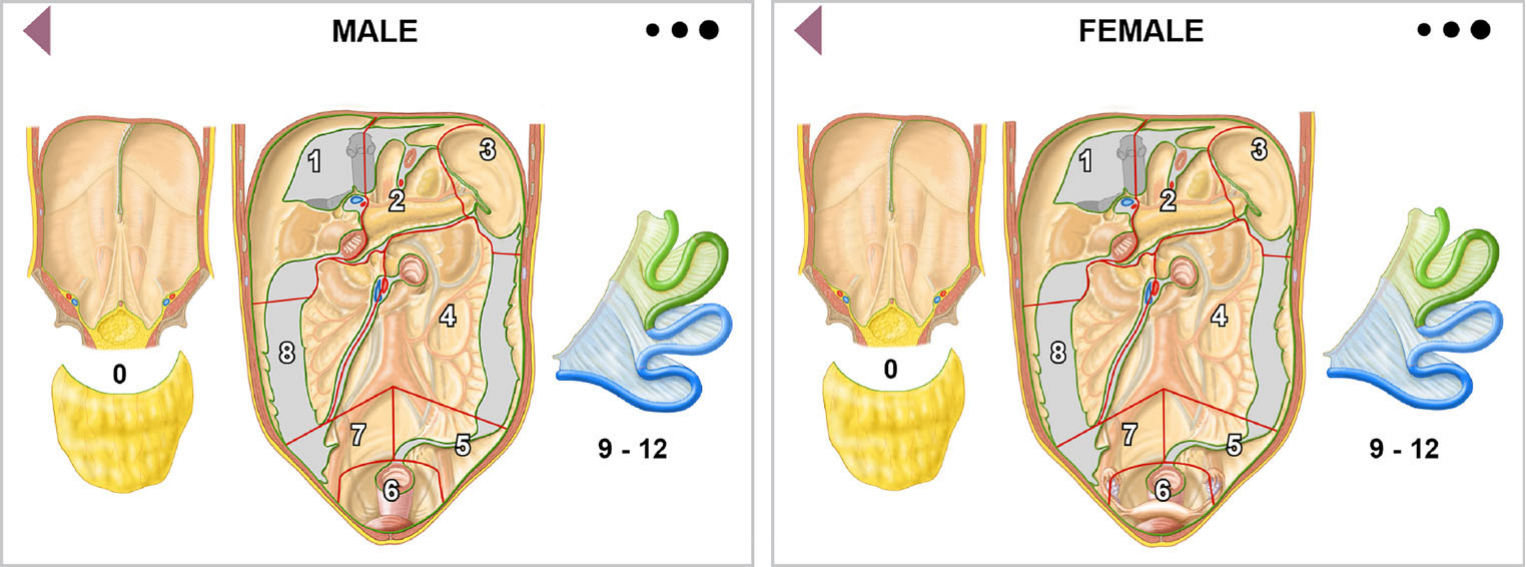
New regional topographic scheme (male/female) suggested for computing PCI and CC-s. *PCI* Peritoneal cancer index, *CC-s* completeness of cytoreduction score

**Fig. 3:**
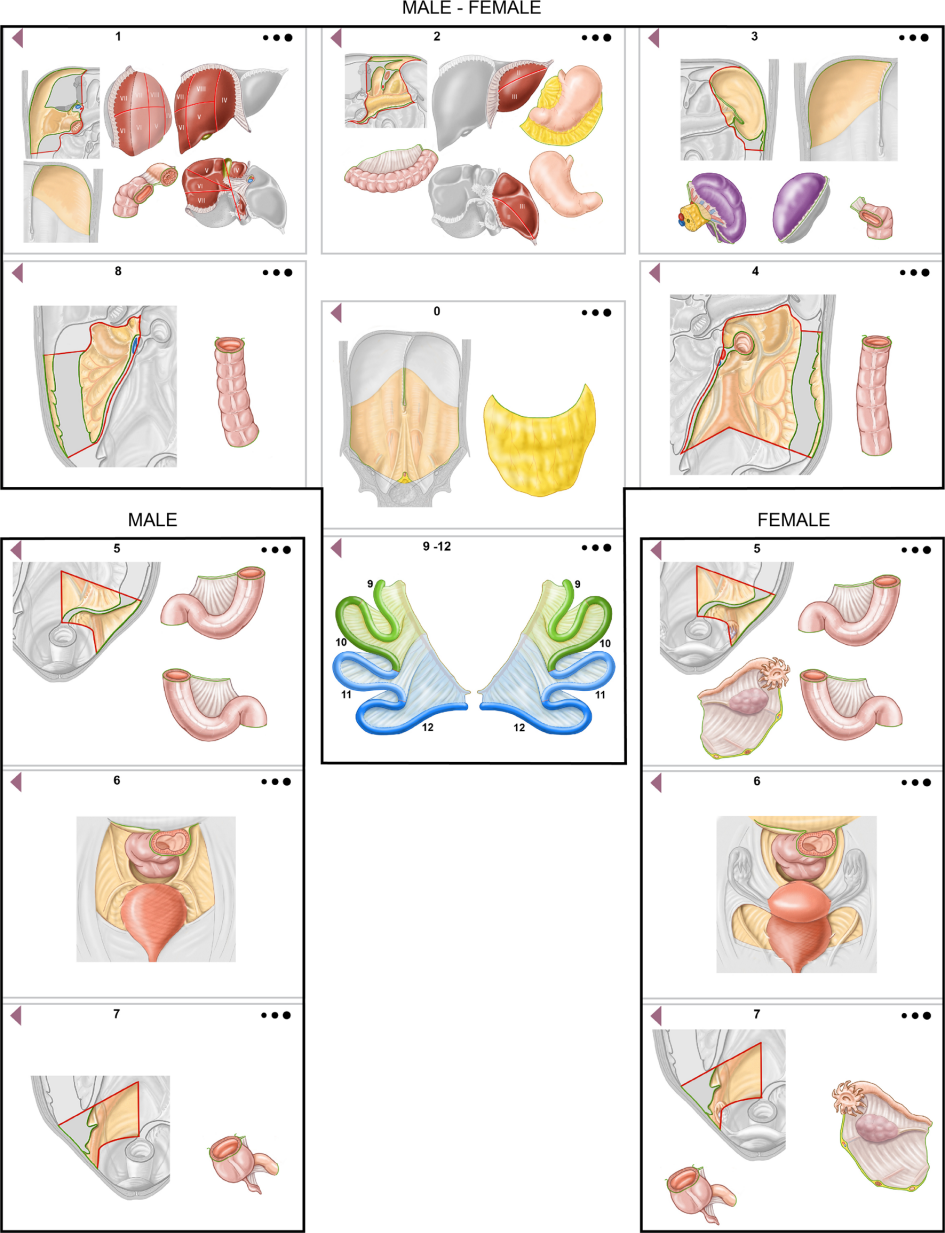
Organs and anatomic structures contained in each region

Based on the classic, graphical black and white representation for assessing the PCI in PSMs, we developed new anatomic models for males and females, illustrating the patient’s real anatomy in color, allowing a three-dimensional image effect, and maintaining, as far as possible, Sugarbaker’s concepts, including clockwise numbering, total number of regions, and lesion size score criteria, thus creating a topographic scheme (Fig. 2). The same new model, always according to Sugarbaker’s lesion size score criteria, served to localize and quantify residual disease and calculate the CC-s after CRS. To avoid structural overlap, we indicated the abdominal wall as region 0, comprising the greater omentum anatomically overlying the abdominal organs. We defined the borders demarcating the various endoabdominal regions unequivocally according to anatomic landmarks: falciform ligament, gastrosplenic ligament, transverse mesocolon, mesenteric root, iliac axes, and pelvic inlet. The concepts used for identifying regions 9–12 (the upper and lower jejunum and ileum) remained unchanged. We illustrated in color the specific organs and structures contained in each region (Fig. 3). Regions 1–3 comprised the organs and structures between the transverse mesocolon and diaphragmatic domes; two landmarks (the falciform ligament and gastrolienal ligament) divided the regions longitudinally. Region 1 included the upper surface of the right liver lobe, the undersurface of the right hemidiaphragm, the gallbladder and hepatic pedicle, the first duodenal portion, and right colonic flexure. Region 2 included the left lobe of the liver, anterior and posterior surface of the stomach, transverse colon between the right and left colonic flexure and its mesocolon, lesser omentum and omental bursa with the anterior surface of the pancreas, and portion of the greater omentum between the greater curvature of the stomach and transverse colon. Region 3 included the undersurface of the left hemidiaphragm, pancreatic tail, gastrosplenic ligament, spleen, and the left colonic flexure (Fig. 3). Regions 4 and 8 were demarcated superiorly by the transverse mesocolon, inferiorly by the iliac axes, and laterally by the right and left abdominal gutter. The two regions were divided longitudinally by the mesenteric root, and both regions included the ascending and descending colon and mesocolon; region 4 included the fourth duodenal portion and the Treitz ligament. Regions 5–7 lay in the space between the iliac axes and the pelvic inlet, separated by the only imaginary line joining the aortic bifurcation to the upper boundary of the pelvic inlet. Region 5 included the sigmoid colon and its mesocolon, and, in women, the left ovary and ovarian tube. Region 7 included the cecum, appendix, the last 10 cm of the distal ileum, and, in women, the right ovary and ovarian tube. Region 6, delimited by the pelvic inlet, included the intraperitoneal rectum, the Douglas pouch, the peritoneum covering the bladder, and, in women, the uterus. For calculating the PCI, the scheme for each region contained three light-grey to black circles, differing in diameter and representing the different lesion size scores (LS1, LS2, and LS3) that could be dragged to the involved anatomic structure so that the computerized system calculated the final score. Similarly, for calculating the CC-s, each region contained three light-grey to black circles, differing in diameter and representing the size of residual disease in a specific region (CC1, CC2, and CC3). The PSM staging system also allowed the user, while dragging the three circles for calculating the PCI or CC-s, to specify, exclusively for LS1/2 and CC1/2, the number of presurgical, perioperative, or residual cancerous implants in each region.

## Web Application Technical Specifications

The PSM staging system is a web-based application accessible through a network that uses a standard web browser as the user terminal and therefore runs on traditional network protocols. To ensure fully secure data management, a data encryption protocol allows safe communication from the source to the recipient (end-to-end) in transfer control protocol/internet protocol (TCP/IP) networks supplying authentication, data integrity, and full data transport-level encryption. The PSM staging system is written in a cross-platform language (HTML5), with the user’s interface code specified in remote. This solution substantially affects functioning; the major advantage is that the web application has no influence whatsoever on the device’s memory capacity or on its ability to calculate data, given that the core processor and user’s interface are on a remote server. Through their supplied password, authenticated users, once connected to the system, will be able to input data for new patients or update their pre-existing data. Using a simple drag-and-drop system, the user will be able to indicate the extent, site, and number of malignant implants present at diagnosis and therapy, or eventually as residual lesions after surgery, positioning them within the anatomic model, and the system will record all the data inserted on cloud. All data inserted can be visualized and compared, and also exported as a PDF document in a completely anonymous manner. No recorded information will in any way be traceable back to the patient. The system will be optimized for use on a tablet (including iPad, Galaxy Tab, Xperia, and Nexus 9) or computer [personal computer (PC), Apple], and updated to run on all the latest-generation web browsers.

## Discussion

Our computerized, web application for staging PSM fulfills an unmet need for a modern, shareable, comprehensive, user-friendly staging system for a life-threatening, although in selected cases nowadays, treatable disease. Our web-based system has another advantage; it collects the main information for each patient, including clinical features (performance status, histology, disease settings), extent of disease at diagnosis and at surgery (PCI), and residual disease after surgery (CC-s), and distinguishes these features according to the various PSM origins.

The colored images with a three-dimensional effect in our anatomic model also help to describe more accurately the spatial extent of PSM. Having the most precise information possible on the extent and sites involved by malignant spread is an essential requisite for planning therapy and assessing outcome.

Despite keeping Sugarbaker’s original concepts, subdividing the various abdominal regions according to real rather than imaginary boundaries, and describing them in detail, as well as including the user-friendly drag-and-drop feature, makes computing the PCI an easier task and should provide more reliable findings. The new subdivision, distinguishing the abdominal wall apart from the other regions, received strong support from publications, indicating abdominal wall disease as being responsible for high postoperative morbidity, and identifying it as an independent prognostic variable.^17^,^18^ Visualizing the various abdominal regions in detail allows the physician to far more precisely localize malignant disease in each anatomic site, thus defining the relationship between the organs and structures involved and outcome, a problem so far addressed only for the small bowel.^10^ In general, these advantages could also help to overcome the reported drawbacks in Sugarbaker’s original PCI.^16^,^19^,^20^ These new features, as well as the drag-and-drop tool, should make it easier to compare the PCI computed in the preoperative setting from diagnostic imaging or at laparoscopy with the PCI computed by the surgeon at surgical exploration, thus improving the patient selection criteria. Although the main, currently used imaging techniques [computed tomography (CT), magnetic resonance imaging (MRI), and positron emission tomography (PET)/CT] achieve high reliability indexes when undertaken by radiologists experienced in this field, intraoperative assessment tends to disclose malignant implants that diagnostic imaging before CRS has missed or underestimated, especially in specific abdominal regions.^21^,^22^ The problem on how to select patients with PSM to undergo surgery acquires especial importance in patients with PSM from colorectal and gastric cancer, given the reportedly high incidence of open–close procedures.^14^,^23^

A distinctive innovation in our PSM staging system is that for LS1 and LS2 we explicitly specify the number of lesions found in a given abdominal region. Although these data leave the PCI classification unchanged, knowing the number of lesions present in each region could provide important, previously unavailable information that could help guide therapy and indicate the patient’s outcome.

By using the same anatomic model and specifying the size, site, and number of residual lesions after CRS, our digital tool addresses the major, so far under-investigated problem related to the completeness of cytoreduction.^24^–^26^ Despite the strict selection criteria usually applied in the more accredited experienced centers, and regardless of the type of PSM, minimal residual disease is a frequent event in patients treated with CRS plus HIPEC and is directly proportional to the PCI.^26^ Even though minimal residual disease (CC1), with the exception of PSMs from gastric cancer,^13^,^26^ is usually considered an acceptable result after CRS plus HIPEC, the outcome depends on several variables, including the biologic aggressiveness of the original PSM, perfusion variables, and chemosensitivity.^27^,^28^ Given that survival rates differ significantly between CC0 and CC1 for PSM of gastric or colorectal origin, and differ less in mesothelioma and pseudomyxomas,^3^,^6^,^13^,^29^ the completeness of cytoreduction is a dynamic concept. Whenever CC0 and CC1 scores yield minor differences in survival, our tool can supply extra, previously unavailable information about the number and sites of residual disease, thus stratifying patients into outcome classes, as others have already suggested for peritoneal mesothelioma.^30^,^31^ In patients with minimal residual disease, our digital tool can therefore provide indications for iterative CRS plus HIPEC,^32^,^33^ and help in planning further chemotherapy sessions.

Among the possible limitations related to our computerized PSM staging system is convincing those who are used to applying conventional staging classifications and concentrating their efforts on therapy to accept and use a modern digital anatomic tool. These drawbacks will eventually become evident as the surgical groups involved in treating PSM begin to use our online web application. An immediate future direction is to update our anatomic PSM staging system according to the latest research on integrated anatomic–biologic prognostic models.^20^,^34^,^35^

## Electronic Supplementary Material

Below is the link to the electronic supplementary material.

Supplementary material 1 (MP4 13879 kb)

Supplementary material 2 (MP4 28459 kb)

Supplementary material 3 (MP4 8818 kb)

## References

[1] Sugarbaker PH (1996). Peritoneal carcinomatosis: principle of management.

[2] Sugarbaker PH (2013). Cytoreductive surgery and perioperative chemotherapy for peritoneal surface malignancy. Textbook and video atlas.

[3] Chua TC, Moran BJ, Sugarbaker PH, Levine EA, Glehen O, Gilly FN (2012). Early- and long-term outcome data of patients with pseudomyxoma peritonei from appendiceal origin treated by a strategy of cytoreductive surgery and hyperthermic intraperitoneal chemotherapy. J Clin Oncol..

[4] Bakrin N, Bereder JM, Decullier E, Classe E, Msika JM, Lorimier G (2013). Peritoneal carcinomatosis treated with cytoreductive surgery and hyperthermic intraperitoneal chemotherapy (HIPEC) for advanced ovarian carcinoma: a French multicentre retrospective cohort study of 566 patients. Eur J Surg Oncol..

[5] Yang XJ, Huang CQ, Suo T, Mei LJ, Yang GL, Cheng FL (2011). Cytoreductive surgery and hyperthermic intraperitoneal chemotherapy improves survival of patients with peritoneal carcinomatosis from gastric cancer: final results of a phase III randomized clinical trial. Ann Surg Oncol..

[6] Elias D, Gilly F, Boutitie F, Quenet F, Bereder JM, Mansvelt B (2010). Peritoneal colorectal carcinomatosis treated with surgery and perioperative intraperitoneal chemotherapy: retrospective analysis of 523 patients from a multicentric French study. J Clin Oncol..

[7] Yan TD, Deraco M, Baratti D, Kusamura S, Elias D, Glehen O (2009). Cytoreductive surgery and hyperthermic intraperitoneal chemotherapy for malignant peritoneal mesothelioma: multi-institutional experience. J Clin Oncol..

[8] Jacquet P, Sugarbaker PH (1996). Clinical research methodologies in diagnosis and staging of patients with peritoneal carcinomatosis. Cancer Treat Res..

[9] da Silva RG, Sugarbaker PH (2006). Analysis of prognostic factors in seventy patients having a complete cytoreduction plus perioperative intraperitoneal chemotherapy for carcinomatosis from colorectal cancer. J Am Coll Surg..

[10] Elias D, Mariani A, Cloutier AS, Blot F, Goéré D, Dumont F (2014). Modified selection criteria for complete cytoreductive surgery plus HIPEC based on peritoneal cancer index and small bowel involvement for peritoneal carcinomatosis of colorectal origin. Eur J Surg Oncol..

[11] Goéré D, Souadka A, Faron M, Cloutier AS, Viana B, Honoré C (2015). Extent of colorectal peritoneal carcinomatosis: attempt to define a threshold above which HIPEC does not offer survival benefit: a comparative study. Ann Surg Oncol..

[12] Canbay E, Mizumoto A, Ichinose M, Ishibashi H, Sako S, Hirano M (2014). Outcome data of patients with peritoneal carcinomatosis from gastric origin treated by a strategy of bidirectional chemotherapy prior to cytoreductive surgery and hyperthermic intraperitoneal chemotherapy in a single specialized center in Japan. Ann Surg Oncol..

[13] Yonemura Y, Elnemr A, Endou Y, Ishibashi H, Mizumoto A, Miura M (2012). Effects of neoadjuvant intraperitoneal/systemic chemotherapy (bidirectional chemotherapy) for the treatment of patients with peritoneal metastasis from gastric cancer. Int J Surg Oncol..

[14] Goéré D, Malka D, Tzanis D, Gava V, Boige V, Eveno C (2013). Is there a possibility of a cure in patients with colorectal peritoneal carcinomatosis amenable to complete cytoreductive surgery and intraperitoneal chemotherapy?. Ann Surg..

[15] Di Giorgio A, Naticchioni E, Biacchi D, Sibio S, Accarpio F, Rocco M (2008). Cytoreductive surgery (peritonectomy procedures) combined with hyperthermic intraperitoneal chemotherapy (HIPEC) in the treatment of diffuse peritoneal carcinomatosis from ovarian cancer. Cancer..

[16] Harmon RL, Sugarbaker PH (2005). Prognostic indicators in peritoneal carcinomatosis from gastrointestinal cancer. Int Semin Surg Oncol..

[17] Nunez MF, Sardi A, Jimenez W, Nieroda C, Sittig M, MacDonald R (2015). Port-site metastases is an independent prognostic factor in patients with peritoneal carcinomatosis. Ann Surg Oncol..

[18] Nunez MF, Sardi A, Nieroda C, Jimenez W, Sittig M, MacDonald R (2015). Morbidity of the abdominal wall resection and reconstruction after cytoreductive surgery and hyperthermic intraperitoneal chemotherapy (CRS/HIPEC). Ann Surg Oncol..

[19] Swellengrebel HA, Zoetmulder FA, Smeenk RM, Antonini N, Verwaal VJ (2009). Quantitative intra-operative assessment of peritoneal carcinomatosis: a comparison of three prognostic tools. Eur J Surg Oncol..

[20] Verwaal VJ, van Tinteren H, van Ruth S, Zoetmulder FA (2004). Predicting the survival of patients with peritoneal carcinomatosis of colorectal origin treated by aggressive cytoreduction and hyperthermic intraperitoneal chemotherapy. Br J Surg..

[21] Esquivel J, Chua TC, Stojadinovic A, Melero JT, Levine EA, Gutman M (2010). Accuracy and clinical relevance of computed tomography scan interpretation of peritoneal cancer index in colorectal cancer peritoneal carcinomatosis: a multi-institutional study. J Surg Oncol..

[22] Torkzad MR, Casta N, Bergman A, Ahlström H, Påhlman L, Mahteme H (2015). Comparison between MRI and CT in prediction of peritoneal carcinomatosis index (PCI) in patients undergoing cytoreductive surgery in relation to the experience of the radiologist. J Surg Oncol..

[23] van Oudheusden TR, Braam HJ, Luyer MD, Wiezer MJ, van Ramshorst B, Nienhuijs SW (2015). Peritoneal cancer patients not suitable for cytoreductive surgery and HIPEC during explorative surgery: risk factors, treatment options, and prognosis. Ann Surg Oncol..

[24] Stoeckle E, Paravis P, Floquet A, Thomas L, Tunon de Lara C, Bussières E (2004). Number of residual nodules, better than size, defines optimal surgery in advanced epithelial ovarian cancer. Int J Gynecol Cancer..

[25] Kubler S, Jähne J, Ceelen WP (2007). Staging and scoring of peritoneal carcinomatosis. Peritoneal carcinomatosis: a multidisciplinary approach.

[26] González-Moreno S, Kusamura S, Baratti D, Deraco M (2008). Postoperative residual disease evaluation in the locoregional treatment of peritoneal surface malignancy. J Surg Oncol..

[27] Van der Speeten K, Stuart OA, Sugarbaker PH (2012). Pharmacology of perioperative intraperitoneal and intravenous chemotherapy in patients with peritoneal surface malignancy. Surg Oncol Clin N Am..

[28] Lambert LA (2015). Looking up: recent advances in understanding and treating peritoneal carcinomatosis. CA Cancer J Clin..

[29] Baratti D, Kusamura S, Cabras AD, Bertulli R, Hutanu I, Deraco M (2013). Diffuse malignant peritoneal mesothelioma: long-term survival with complete cytoreductive surgery followed by hyperthermic intraperitoneal chemotherapy (HIPEC). Eur J Cancer..

[30] Baratti D, Kusamura S, Cabras AD, Dileo P, Laterza B, Deraco M (2009). Diffuse malignant peritoneal mesothelioma: failure analysis following cytoreduction and hyperthermic intraperitoneal chemotherapy (HIPEC). Ann Surg Oncol..

[31] Schaub NP, Alimchandani M, Quezado M, Kalina P, Eberhardt JS, Hughes MS (2013). A novel nomogram for peritoneal mesothelioma predicts survival. Ann Surg Oncol..

[32] Chua TC, Quinn LE, Zhao J, Morris DL (2013). Iterative cytoreductive surgery and hyperthermic intraperitoneal chemotherapy for recurrent peritoneal metastases. J Surg Oncol..

[33] Ihemelandu C, Bijelic L, Sugarbaker PH (2015). Iterative cytoreductive surgery and hyperthermic intraperitoneal chemotherapy for recurrent or progressive diffuse malignant peritoneal mesothelioma: clinicopathologic characteristics and survival outcome. Ann Surg Oncol..

[34] Esquivel J, Lowy AM, Markman M, Chua T, Pelz J, Baratti D (2014). The American Society of Peritoneal Surface Malignancies (ASPSM) multiinstitution evaluation of the Peritoneal Surface Disease Severity Score (PSDSS) in 1,013 patients with colorectal cancer with peritoneal carcinomatosis. Ann Surg Oncol..

[35] Cashin PH, Graf W, Nygren P, Mahteme H (2012). Patient selection for cytoreductive surgery in colorectal peritoneal carcinomatosis using serum tumor markers: an observational cohort study. Ann Surg..

